# Molecular Evidence of *Pneumocystis* Transmission in Pediatric Transplant Unit

**DOI:** 10.3201/eid1102.040820

**Published:** 2005-02

**Authors:** Britta Höcker, Constanze Wendt, Aimable Nahimana, Burkhard Tönshoff, Philippe M. Hauser

**Affiliations:** * University Children's Hospital, Heidelberg, Germany;; † Hygiene Institute Heidelberg, Germany;; ‡ University Hospital of Lausanne, Lausanne, Switzerland

**Keywords:** Pneumocystis jirovecii, pneumonia, PCP, pediatric renal transplantation, single-strand conformation polymorphism, inter-human transmission, dispatch

## Abstract

We describe an outbreak of *Pneumocystis jirovecii* pneumonia in a pediatric renal transplant unit, likely attributable to patient-to-patient transmission. Single-strand conformation polymorphism molecular typing showed that 3 affected patients had acquired the same 2 strains of *Pneumocystis*, which suggests interhuman infection. An infant with mitochondriopathy was the probable index patient.

Despite intensive medical treatment, *Pneumocystis jirovecii* pneumonia (PCP) is still a severe disease in immunocompromised patients, with a high death rate of up to 50% (*1*). The first report of human *Pneumocystis* infection appeared in 1909; nevertheless, its epidemiology is poorly understood to date. In the 1950s, reports on PCP epidemics in malnourished infants in hospitals and orphanages aroused suspicion of interhuman transmission. In addition, animal studies have demonstrated airborne transmission of *Pneumocystis* (*2*). A case-control study conducted for a cluster of 5 PCP cases in transplant recipients suggested transmission of *P. jirovecii* from AIDS patients to other immunosuppressed persons (*3*). However, molecular typing methods for *P. jirovecii* were lacking so that patient-to-patient transmission could not be assessed at the molecular level. When such techniques were developed in the 1990s, 3 analyses showed different *P. jirovecii* genotypes within clusters (*4**–**6*). A recent analysis at the molecular level of a cluster of 10 PCP cases strongly suggested that HIV-infected persons with active PCP transmitted *P. jirovecii* to renal transplant recipients (*7*). The role of interhuman transmission of *P. jirovecii* in the epidemiology of PCP is still unclear.

## The Outbreak

Having observed no occurrence of PCP in our pediatric renal transplant unit for the last 20 years and only 1 case in all German pediatric renal transplant units during the last 10 years, we encountered 3 consecutive incidents of PCP during a 5-month period. The first patient was a 13-year-old girl, who had received her second renal graft because of cystic kidney disease; PCP developed 4 months after transplantation. The second patient, a 14-year-old boy, fell ill in the ninth posttransplant month; he had bilateral vesicoureteral reflux as underlying renal disease. The third patient was a 13-year-old girl, who had a transplant 2 years before contracting PCP because of cystic renal dysplasia occurring in the context of Bardet-Biedl syndrome.

All 3 children had been given cyclosporine A (average dose 6.7 mg/kg/day), mycophenolate mofetil (1,060 mg/m^2^/day), and methylprednisolone (3.2 mg/m^2^/day), as maintenance immunosuppression. One patient had also received induction therapy with the interleukin-2-receptor-antibody basiliximab. All 3 children had been treated with methylprednisolone pulses for acute rejection episodes 2, 3, and 15 months before PCP was diagnosed.

Clinically, all patients showed nonspecific symptoms, such as mild fever, dyspnea, and dry cough in the absence of auscultatoric anomalies. Laboratory tests showed an elevation of lactic dehydrogenase activity, C-reactive protein concentration in blood, and pronounced hypercalcemia (2.7–3.5 mmol/L), which was interpreted as an extrarenal production of 1.25-dihydroxyvitamin D_3_ by activated alveolar macrophages. We found a significant reduction of S-adenosylmethionine concentration in plasma (6 nmol/L; normal range 86–128 nmol/L), which appears to be specific to PCP, unlike bacterial or other atypical pneumonias (*8*). We measured the blood count of CD4^+^ and CD4/DR^+^T lymphocytes in the third patient to indicate the degree of immunosuppression, since antirejection therapy had been administered 15 months before the occurrence of PCP. The number of CD4^+^T cells was normal at the time of PCP diagnosis (1,100 cells/μL; normal range 505–1,151 cells/μL), while the number of activated T-helper cells was slightly decreased (24 CD4/DR^+^cells/μL; normal range 29–87/μL). Only in the course of PCP did the numbers of CD4^+^ and CD4/DR^+^T lymphocytes drop significantly (308 CD4^+^T cells/μL and 8 CD4/DR^+^cells/μL). Chest radiographs and thorax computed tomographic scans of the 3 children showed typical signs of interstitial pneumonia, e.g., ground-glass opacity.

Diagnosis of PCP was confirmed by the presence of cysts and vegetative forms in bronchoalveolar lavage fluid, proved by immunofluorescence staining, and through detection of *Pneumocystis* DNA by means of polymerase chain reaction (PCR). In spite of intensive antimicrobial therapy, 2 of our 3 renal transplant patients died, at 10 and 28 days, respectively, after the onset of PCP.

To determine if PCP could have been caused by patient-to-patient transmission, we closely examined the course of PCP in the infected children (Figure). Patient 1 stayed on the same ward and same floor, but not in the same room (distance between the rooms' doors ≈10 m), as an infant with a yet-unclassified mitochondriopathy and pneumonia, which later was diagnosed as PCP. Patient 2's hospital stay overlapped that of patient 1; the patients were on the same ward and same floor, in rooms with doors separated by 8 m, before the onset of PCP. Patient 3 spent her holiday with patient 2 in a summer camp organized by our Pediatric Nephrology Division.

**Figure:**
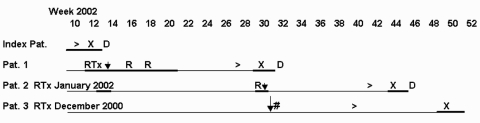
Course of *Pneumocystis* pneumonia (PCP) in 3 pediatric renal transplant recipients and 1 infant suffering from a yet-unclassified mitochondriopathy. R, acute rejection episode; RTx, renal transplantation; ↓, contact; #, ,joint holiday; >, start of PCP symptoms; ▬, hospitalization; X, diagnosis of PCP; D, death.

Proceeding on the assumption that PCP in the 4 children resulted from patient-to-patient transmission, we investigated the genotypes of *P. jirovecii* with the multitarget single-strand conformation polymorphism (SSCP) method. This typing procedure is based on the amplification by PCR of 4 variable regions of the genome, followed by the detection of polymorphisms by means of SSCP. These 4 genomic regions are as follows: internal transcribed spacer number 1 of the nuclear rRNA genes operon (ITS1), the intron of the nuclear 26S rRNA gene (26S), the variable region of the mitochondrial 26S rRNA gene (mt26S), and the region surrounding intron number 6 of the β-tubulin gene (β-tub). Typing procedures were carried out as described elsewhere (*9*). A variable region amplified from a bronchoalveolar lavage fluid specimen can generate either 2 bands (simple pattern) or >2 bands (complex pattern). While a simple pattern corresponds to a single allele of the genomic region, the presence of >2 bands (complex pattern) indicates the existence of several alleles for a given region, most probably attributable to coinfection with multiple *P. jirovecii* types (*10*).

Our analysis showed that all 3 renal transplant patients had acquired the same 2 strains of *Pneumocystis*, types 1 and 2. The infant with mitochondriopathy had been infected with >2 strains, which possibly included types 1 and 2 (Table). In contrast, 3 unrelated cases in patients (patients 4, 5, and 6) from the same hospital harbored other *P. jirovecii* types. The index of discrimination of the method was high (0.93) (*10*), and the probability that patients 1, 2, and 3 were infected with the same strains by chance is extremely low. We observed that the proportion of coinfecting strains within the clinical specimen was more important than the amount of template DNA to detect or not detect a strain by means of the SSCP typing method. A coinfecting strain has to represent 11% of the population to be detected (*11*). Coinfecting strains are missed when very low amounts of template DNA are used, sometimes resulting in a negative PCR; however, this was not the case for the specimens analyzed in the present study.

**Table:** *Pneumocystis jirovecii* genotyping by PCR-SSCP of 4 genomic regions*

Patient	Date	SSCP pattern				
		ITS1	26S	mt26	β-tub	*P. jirovecii* type
Index case-patient	3/27/02	A	A, **B**	**A**, B,C	A, **B**	>2 types (nonidentifiable)
1	7/26/02	A	A, **B**	A	A	**1**, 2
2	10/30/02	A	A, **B**	A	A	**1**, 2
3	12/3/02	A	A, **B**	A	A	**1**, 2
4	2/7/03	A, B	**A**, **B**, C	B, **C**	B, **C**	>2 types (nonidentifiable)
5	2/15/03	A	A, **B**	C	A	6, **44**
6	2/18/03	A, **B**	A, **B**	A	C	**45**, 46

*Bold letters signify the most abundant simple pattern with the complex one, as shown by silver staining. Bold numbers signify the most abundant *P. jirovecii* type. PCR, polymerase chain reaction; SSCP, multitarget single-strand conformation polymorphism.

The index patient had more strains than the other patients (Table) but generated smaller amounts of PCR products with the 4 different PCR tests used, which suggests a lower amount of DNA template. Although excluding a common source of *P. jirovecii* is difficult, the results strongly suggest that all 3 kidney transplant recipients had infected each other, and the infant could have acted as index patient. This transmission may have occurred directly from 1 patient to another but also indirectly through immunocompetent carriers. Indeed, carriage of *P. jirovecii* DNA in the nose of immunocompetent relatives and healthcare workers in close contact with a PCP patient has been described (*12*).

## Conclusions

To our knowledge, this report is the first published on an outbreak of PCP in a pediatric renal transplant unit, probably attributable to patient-to-patient transmission. However, we cannot exclude that the cases described were infected by the same environmental source. The presence of *P. jirovecii* in the air of hospital corridors has been described (*13*), making an environmental reservoir in the hospital possible. Other potential sources of *P. jirovecii* could be asymptomatic *P. jirovecii* carriers, such as immunosuppressed patients (*14**,**15*). Our findings at the molecular level suggest that *P. jirovecii* may be transmitted nosocomially and be acquired by immunosuppressed pediatric transplant recipients. The incubation periods of *P. jirovecii* infection (17, 15, and 19 weeks for patients 1, 2, and 3, respectively) would be longer than those (2–12 weeks) suggested by the previously described clusters of PCP (*3**,**7**,**16*). This finding may reflect a difference between adults and children.

Until the outbreak of PCP outlined in this article, pediatric renal transplant recipients in our hospital and other pediatric renal transplant units in Germany were not given PCP prophylaxis routinely because of possible side effects, such as a rise of serum creatinine values, myelosuppression, and Lyell syndrome. We had not observed any case of PCP in our transplant recipients for the last 20 years without prophylaxis. In the light of the high death rate for PCP, prophylactic treatment with trimethoprim-sulfamethoxazole is highly recommended for the first 6 posttransplant months and during the 4 months after antirejection therapy, in accordance with the guidelines for adults (*1*). According to these guidelines, patient 3, in whom PCP developed 15 months after steroid pulse therapy, would not have been protected by prophylaxis. Whether prophylaxis should be given for a longer period of time remains unknown, particularly since immunosuppression did not appear to be intensive in this patient at the onset of PCP, as indicated by the normal CD4^+^ T-lymphocyte count in peripheral blood and the only slightly decreased number of activated T-helper cells.
